# Comparison of Hybrid Classifiers for Crop Classification Using Normalized Difference Vegetation Index Time Series: A Case Study for Major Crops in North Xinjiang, China

**DOI:** 10.1371/journal.pone.0137748

**Published:** 2015-09-11

**Authors:** Pengyu Hao, Li Wang, Zheng Niu

**Affiliations:** 1 The State Key Laboratory of Remote Sensing Science, Institute of Remote Sensing and Digital Earth, Chinese Academy of Sciences, Beijing, 100101, China; 2 University of Chinese Academy of Sciences, Beijing, 100049, China; University of Calgary, CANADA

## Abstract

A range of single classifiers have been proposed to classify crop types using time series vegetation indices, and hybrid classifiers are used to improve discriminatory power. Traditional fusion rules use the product of multi-single classifiers, but that strategy cannot integrate the classification output of machine learning classifiers. In this research, the performance of two hybrid strategies, multiple voting (M-voting) and probabilistic fusion (P-fusion), for crop classification using NDVI time series were tested with different training sample sizes at both pixel and object levels, and two representative counties in north Xinjiang were selected as study area. The single classifiers employed in this research included Random Forest (RF), Support Vector Machine (SVM), and See 5 (C 5.0). The results indicated that classification performance improved (increased the mean overall accuracy by 5%~10%, and reduced standard deviation of overall accuracy by around 1%) substantially with the training sample number, and when the training sample size was small (50 or 100 training samples), hybrid classifiers substantially outperformed single classifiers with higher mean overall accuracy (1%~2%). However, when abundant training samples (4,000) were employed, single classifiers could achieve good classification accuracy, and all classifiers obtained similar performances. Additionally, although object-based classification did not improve accuracy, it resulted in greater visual appeal, especially in study areas with a heterogeneous cropping pattern.

## Introduction

Crop-type information is important for the global food security system, and there is an urgent demand for accurate crop classification data [1–3]. Since the crop calendar varies among different crops, phenology is the basis of crop classification [4]. Vegetation indices (VI), which could be calculated from remote sensing images, can measure vegetation coverage, and VI time series can describe crop phenology [5–7]. Thus, VI time series have been employed widely to produce crop classification data [8–12]. In addition, the Normalized Difference Vegetation Index (NDVI) has the higher importance score than other features, such as multi-spectral bands, calculated indices and ancillary data [13].

Coarse spatial resolution data, such as Moderate Resolution Imaging Spectroradiometer (MODIS) (250–500m) data, are characterized by high temporal resolution and have shown potential for identifying crops [14, 15]. However, one drawback is that the spatial resolution of MODIS data is relatively coarse, and the classification accuracy is affected by mixed pixels when the field size is small. For sensors at finer spatial resolution, such as Landsat-5/7 TM/ETM+ (at 30-m resolution), the revisit period is relatively long (e.g., 16days for Landsat). Thus, Landsat cannot provide enough cloud-free imagery for most regions of the world [16]. As some other sensors (such as Huan Jing and DEIMOS) can provide images at medium spatial resolution [17, 18], the use of multi-sensor merged image time series has improved the performance of crop identification at medium resolution [19, 20].

A range of classifiers, such as Decision Tree (DT), Support Vector Machine (SVM), Random Forest (RF), and C5 [14, 21, 22], have been employed for crop type classification. In addition, totake advantage of single classifiers and increase the classifiers’ discriminatory power, some hybrid classifiers have been proposed based on product and sum rules [23]. For example, Du, Chang [24] mixed Spectral Information Divergence (SID) and Spectral Angle Mapper (SAM) and Ghiyamat and Shafri [25] mixed SID and Spectral Correlation Measure (SCM) utilizing product rules, and the mixed classifiers have shown good performance in hyperspectral classification. However, a drawback is that the improvement of the product rule is limited when multiple (more than three) classifiers are used. Various other hybrid strategies, such as the multi-classifier approaches multiple voting (M-voting) and probabilistic fusion (P-fusion), have been employed to combine the classification results obtained from multi-spectral and textual features and have improved the performance of the classification [26]. However, few studies have tested the performance of multi-classifier systems on integrating classifiers.

Pixel-based image analyses (PBIA) have led to some misclassifications, which are due mainly to 1) the similar spectral characteristics of some crop classes, 2) the spectral variability due to the canopy and bare soil background reflectance and different crop development schedules, and 3) the mixed pixels located at the boundaries between classes [8]. Object-based image analyses (OBIA) were proposed to solve these problems [13], and a number of studies have found that OBIA methods are effective for classification for both high- and moderate-resolution imagery [27]. In addition, several studies have compared the performance of OBIA with that of PBIA. For images with high spatial resolution (spatial resolution better than 10 m), OBIA outperformed PBIA with better classification accuracy and the capacity to extract patch boundaries [28, 29]. At medium spatial resolution (10–100m), OBIA generally provides a visually appealing appearance, but conclusions about statistical accuracy have varied. Some studies have shown that OBIA achieved higher classification accuracy than PBIA [30, 31]; some obtained the opposite conclusion, PBIA obtaining better accuracy [32, 33]; and others concluded that OBIA and PBIA acquired similar classification accuracy [34, 35]. Thus, additional study remains essential to test the potential of OBIA in improving the classification of specific crop classes.

Xinjiang is an important cotton-production region, and the crop fields in North Xinjiang are characterized by large size (larger than 20 ha). Then, two representative counties in North Xinjiang, Bole and Manas, were selected in this research to test the potential of NDVI time series of 30-m spatial resolution to classify crops at both the pixel and object level. Therefore, the objectives of this study were to 1) estimate the potential of NDVI time series obtained by merging Landsat-5 and Huan Jing (HJ)-1 data for crop classification at medium resolution [17]; 2) compare the performance of hybrid strategies (M-voting and P-fusion) with the single classifiers for crop classification; and 3) compare the performance of OBIA and PBIA for crop classification.

## Study Regions and Data Description

### Description of the study area and crop calendar

**Fig 1:**
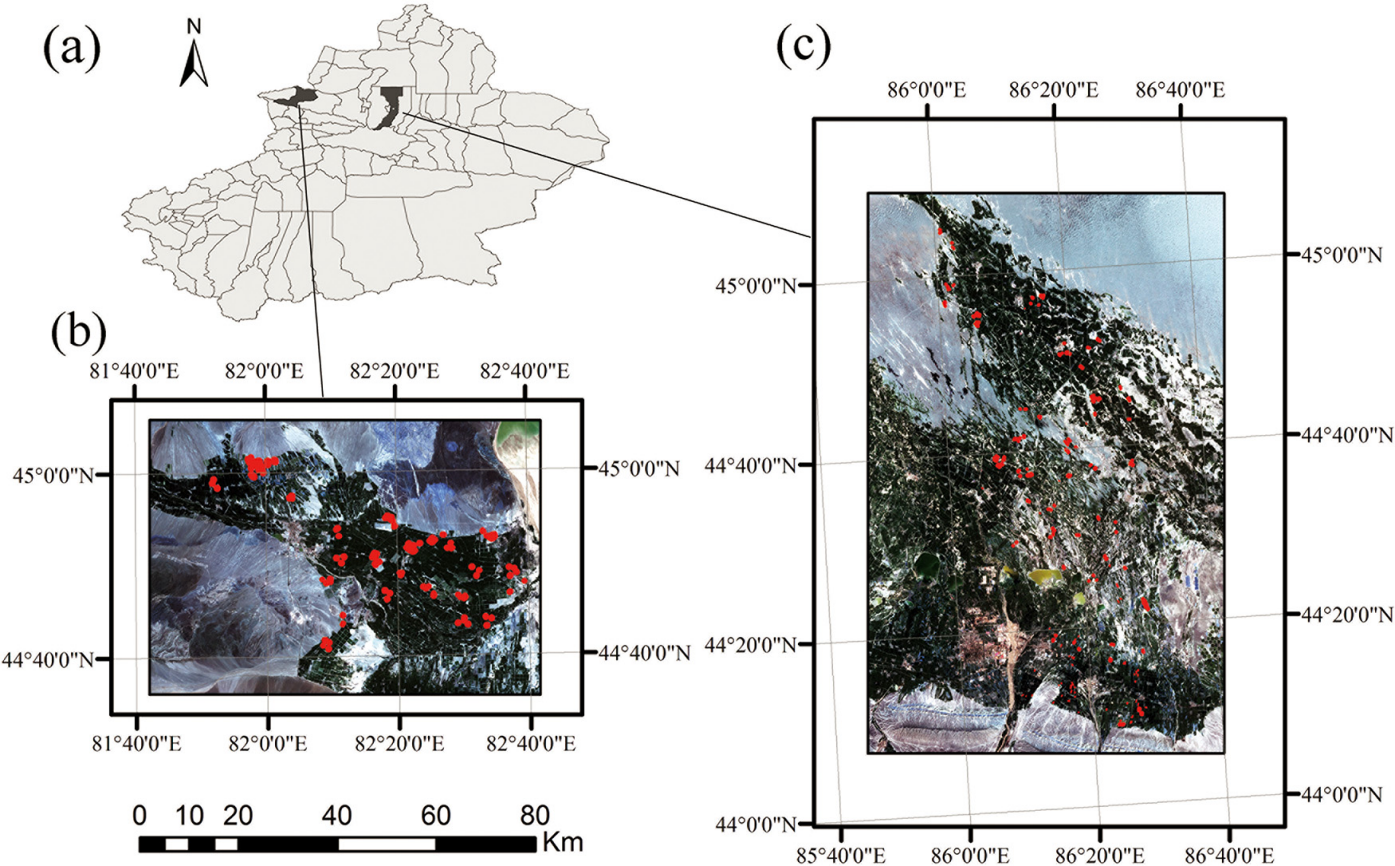
Study areas: (a) Location of Bole and Manas in Xinjiang and extent of Xinjiang. (b) Bole (2011/7/11) and (c) Manas (2011/7/13). The red patterns on the images are distributions of ground reference data.

In this research, we selected two representative agricultural regions in northern Xinjiang: one located in Bole County, which has 32 kha of cropland (44°20′–45°23′N, 80°40′–82°42′E), a second located in Manas County, which covers 180 kha of cropland (43°17′–45°20′N, 85°17′–86°46′E) (Fig 1). The regions have a temperate continental climate characterized by dryness and drought. The annual average temperature and rainfall are 7.0°C and 202mm in Bole and 7.2°Cand 208mm in Manas, respectively.

**Fig 2:**
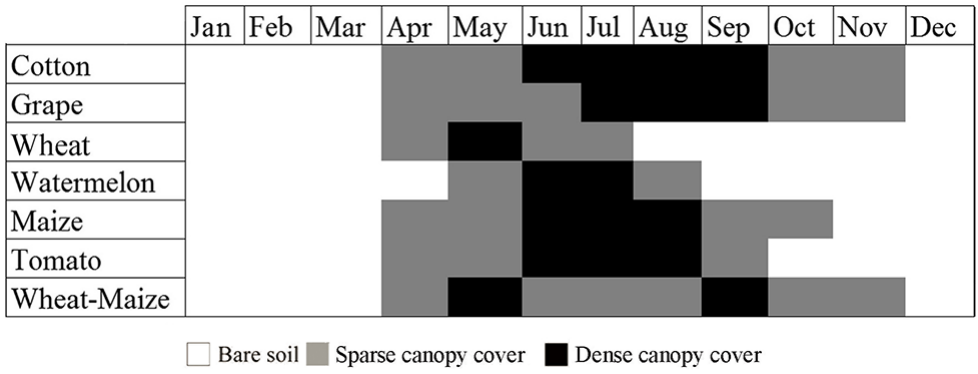
Vegetation cover fraction of crop types within the annual growing season.

The dominant crops grown in the study areas include cotton, maize (spring maize and summer maize), watermelon, grape, tomato, and wheat. The vegetation cover fraction for each crop type over the growing season is presented in Fig 2. Cotton, spring maize, watermelon, tomato, and grape are planted in early April and begin their growth mostly during the June–July period. For harvest, watermelon and tomato are harvested in August, spring maize is harvested in early September, and grapes and cotton are harvested during the August–September and September–October periods. Winter wheat is planted in early November, begins its growth in the next April, and is reaped for harvest in late June. After that, some fields are in rotation, and others are planted to summer crops such as summer maize. Thus, we divided the winter wheat into two classes depending on whether summer crops are planted in the same field or not.

### Datasets

#### Satellite images and vegetation indices

We selected cloud-free (cloud cover less than 20%) satellite images in both study regions covering the entire growing season (Table 1), including both the Landsat TM CDR land surface reflectance product and the Huan Jing (HJ) CCD level 2 product of 2011 [36]. The HJ-1 satellite constellation, launched by the Chinese Government in 2008, has two CCD cameras that observe a broad coverage of 720 km and have spatial resolution of 30m. The CCD cameras have four visible and near-infrared bands, which include B1 (0.43–0.52nm), B2 (0.52–0.60nm), B3 (0.63–0.69nm), and B4 (0.76–0.9nm) [37]. In this research, we intended to obtain a NDVI time series at 30-m spatial resolution and at approximately 15-day temporal resolution during the growing season. However, TM images cannot cover the entire growing season of the study areas at such a high temporal resolution. Therefore, we employed cloud-free Landsat TM and HJ CCD (with similar spatial resolution to Landsat TM) images to build the image time series. All HJ images were georeferenced to the UTM WGS84 zone 44N (Bole) and 45N (Manas), and the HJ images were then registered to the TM images, achieving an RMSE of less than 0.3 pixels using a second-order polynomial transformation and B-linear resampling. Then, radiance calibration and Fast Line-of-sight Atmospheric Analysis of Hypercubes (FLAASH) atmospheric correction were performed using Environment for Visualizing Images (ENVI) for all images [38–40]. NDVI was calculated using the reflectance of visible (red) and near-infrared (NIR) bands for both TM and HJ images using Eq (1).

$$\text{NDVI}=\frac{\rho \;(\text{NIR})-\rho \;(\text{Red})}{\rho \;(\text{NIR})+\rho \;(\text{Red})}$$ (1)

**Table 1:** TM and HJ-1 CCD images for both study regions.

Sensors	Bole	Manas
Landsat-5 TM	4/6, 4/22, 5/24, 7/11, 7/27, 9/13	4/24, 5/10, 6/28, 7/13 10/1
HJ1A-CCD1	6/8, 9/27	
HJ1A-CCD2	10/16	10/27
HJ1B-CCD1	5/10, 8/16	9/16
HJ1B-CCD2		5/28, 8/15

#### Ground reference data

Ground-truth data were obtained by fieldwork in the study regions during 2011. Fields were selected to represent the full variety of crop types and an even distribution across the study areas. The selected fields, 457 fields in Bole and 435 fields in Manas, were then surveyed. For each field, the crop type was collected as attribute information. Field boundaries were recorded using GPS and digitized as polygons in ArcGIS. To avoid boundary pixels, polygons were converted to a raster format using the TM grid. In total, 10,855 sample pixels for Bole and 9,991 pixels for Manas were obtained. The distribution of ground-truth data is shown in Fig 1. The amounts of training and validation samples for each crop type are shown in Table 2, and the training and validation dataset are provided in S1 Dataset.

**Table 2:** Number of training and validation samples.

Study area	Bole			Manas		
	Surveyed fields	Training	Validation	Surveyed fields	Training	Validation
	(Polygons)	(Pixels at 30m)		(Polygons)	(Pixels at 30m)	
Cotton	229	2502	2500	269	2206	2205
Spring Maize	36	308	308	63	977	977
Grape	43	718	718	0	0	0
Wheat	74	686	686	54	839	839
Watermelon	75	695	694	0	0	0
Tomato	0	0	0	49	710	706
Wheat-Summer crop	68	520	520	28	266	266
Total	457	5429	5426	435	4998	4993

## Methods

**Fig 3:**
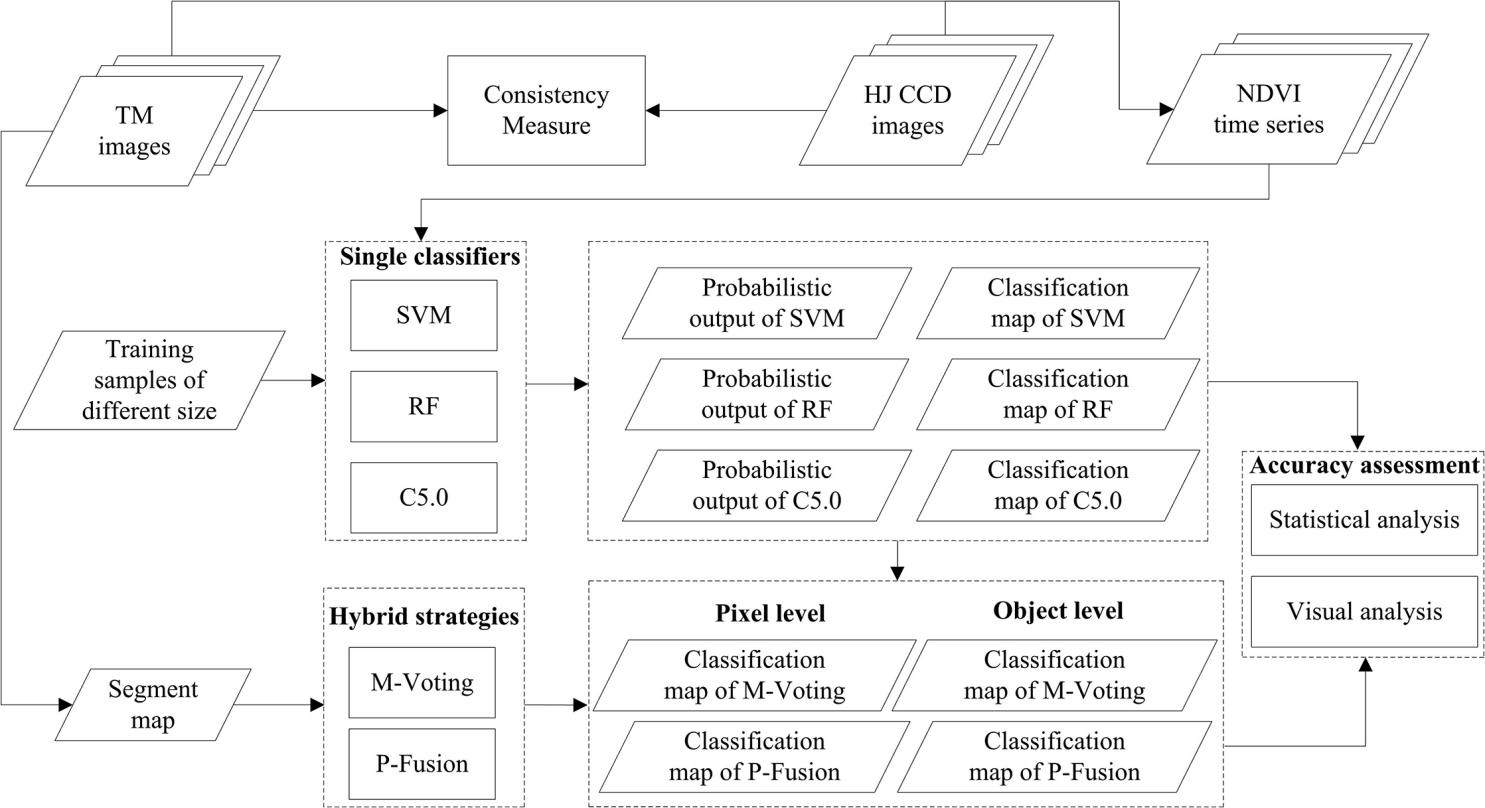
Methodology of the study.

The methodology of the study is presented in Fig 3. First, we measured the consistency between Landsat TM NDVI and HJ CCD NDVI, and then utilized Landsat TM and HJ CCD images to build time series NDVI covering the entire growing season. Then, we used the single classifiers to classify crop types and obtained both a classification map and probabilistic outputs for each classifier. Afterward, the performance of two fusion strategies, M-voting and P-fusion, at both pixel and object levels were assessed using both statistical and visual analyses. The training sample size varied from 50 to 4000 (50, 100, 250, 500, 750, 1000, 1500, 2000, 2500, 3000, 3500, and 4000) in each study area, and for each training sample size, the samples were randomly selected from the training sample set listed in Table 2. Additionally, all classifiers were trained ten times for each training sample size, and both the average and standard deviation of the classification accuracy were reported.

### Similarity Measure between Landsat and HJ NDVI

We evaluated the NDVI consistency between Landsat-5 TM and HJ-1 CCD data by comparing the NDVI of the two sensors for similar dates (Table 3). To reduce the potential impacts of registration inaccuracy, we defined subsets of homogeneous regions of interest (ROI) with 3 × 3 windows located in the middle of larger homogeneous “patches” [41]. Average values of these sampling windows were used to compare the consistency between Landsat-5 TM and HJ-1 CCD NDVI. Through this process, we defined subsets of the ROIs for different crop types, selecting 170 and 153 windows within Bole and Manas, respectively. Scatter plots and linear relationships were used to examine how Landsat-5 TM and HJ-1 CCD NDVI differed in performance.

**Table 3:** Images used for consistency measurement for both study areas.

Sensors	Bole	Manas
Landsat-5 TM	5/24, 7/27, 9/13	5/10, 7/13, 10/1
HJ-1 CCD	5/23, 7/29, 9/14	5/10, 7/13, 10/1

### Image segmentation

To compare the performances of different classifiers at pixel and object level, we segmented the image using the multi-resolution segmentation (MRS) algorithm of eCognition [42]. The MRS algorithm is a “bottom-up” approach that begins with pixel-sized objects and iteratively grows through the pair-wise merging of neighboring objects based on several user-defined parameters, including scale, color/shape, weights of spectral bands, and smoothness/compactness [42]. Landsat images of three time periods were used during the segmentation process in both of the study regions; and a summary of the segmentation parameters used is presented in Table 4.

**Table 4:** Summary of variables and parameters used in the segmentation.

Bole		Manas	
Image	weight	Image	weight
TM(b1, b2, b3, b4)- Apr 22/2013	1	TM(b1, b2, b3, b4)- May 10/2013	1
TM(b1, b2, b3, b4)- Jul 11/2013	1	TM(b1, b2, b3, b4)- Jul 13/2013	1
TM(b1, b2, b3, b4)- Sep 13/2013	1	TM(b1, b2, b3, b4)- Sep 16/2013	1
TM NDVI- Apr 22/2011	2	TM NDVI- May 10/2011	2
TM NDVI- Jul 11/2011	2	TM NDVI- Jul 13/2011	2
TM NDV I-Sep 13/2011	2	TM NDVI- Sep 16/2011	2
Segmentation parameter		Segmentation parameter	
Scale	80	Scale	50
Compactness/Smoothness factor	0.2/0.8	Compactness/Smoothness factor	0.2/0.8
Shape/colour factor	0.5/0.5	Shape/colour factor	0.5/0.5

### Classifiers

#### Single classifiers

The performances of the following three single classifiers were evaluated: 1) Random Forest (RF), 2) Support Vector Machine (SVM), and 3) C 5.0 Rule-Based Model (C5.0). SVM is a non-parametric supervised classifier derived from statistical learning theory [10]. In the simplest form, SVMs are linear binary classifiers that label a given test sample from one of the two possible classes. The classifiers use training samples to find the optimal hyperplanes that separate classes with minimum classification error. Significantly, only the training samples that lie on the margin (called support vectors) are used to define the hyperplanes. The simplest SVMs assume that the problems are linearly separable. However, “data points” of different classes overlap in practice. As a result, the basic linear decision boundaries cannot classify patterns with high accuracy. Thus, the linearly inseparable problems are solved by transforming the nonlinear correlations into a higher (Euclidean or the Hilbert) space using a kernel function [21]. Another problem is that remote sensing classifications always involve multi-class situations. In this case, the binary classifier (simplest SVM) is used as a multi-class classifier based on one-against-one and one-against-others methods [21, 43]. Generally, SVM is not based on the assumption that the data are normally distributed for a particular image; thus, SVM could outperform classifiers based on maximum likelihood theory [10]. Additionally, SVM has the advantages of being able to deal with high-dimensional datasets and small training samples [44–46], and SVM has been utilized widely for crop classification [10, 20, 47]. In this study, implementation of SVM was performed with the libSVM (library e1071 for R) [48].The widely used radial basis function kernel (RBF) was selected, and the data space was normalized to a common scale [0, 1]. In addition, training of the SVM included choosing a kernel parameter *“γ”* (gamma) and a regularization parameter “*C”* (cost). *“C”* controls the penalty associated with misclassified training samples, and *“γ”* determines the gamma of the kernel function [49]. Then, the parameters *“C”* and *“γ”* were selected using a systematic 2-D space spanned by *“γ”*and *“C”*.

The RF algorithm is an ensemble machine learning technique that combines multiple trees [22]. Each tree is constructed using two-thirds of the original cases. Then, the remaining one-third of the cases are employed to generate a test classification, with an error referred to the “out-of-bag error” (OOB error). Subsequently, the model output is determined by the majority vote of the classifier ensemble [50]. Two free parameters can be optimized in the RF algorithm: the number of trees (*ntree*) and the number of features to split the nodes (*mtry*). The advantages of the RF algorithm, such as the relatively high efficiency with large datasets, the probability output for each class, and the generated OOB error (an internal unbiased estimate of the generalization error), make it suitable for remote sensing applications [51]. In this study, both the crop-label and probabilistic output were obtained using the Random Forest library for R [52]. The *“ntree”* parameter was set to a relatively high value of 1000 to allow convergence of the OOB error statistic, and “*mtry”* was set to the square root of the total number of input features [53].

C5.0 is a decision tree algorithm developed from C4.5. In C4.5, when training the model, all training samples are set as the root of the decision tree. Then, the gain information ratio of every feature is calculated based on the entropy of the feature, and the feature with the highest information gain is selected to split the data into multi-subsets. The algorithm repeats this procedure on each subset until all instances in the subset belong to the same class and a leaf node is created [27, 54]. C5.0 advanced C4.5 by the development of a boosting technique (generating and combining multiple classifiers to improve predictive accuracy) [55]. C5.0 has several advantages including that it is fast to train and it has a set of rules. Thus, it has been employed widely in land use and land cover classification (LULC) [56, 57]. In this study, C5.0 was implemented using the library C50 for R [58], and the parameters *“trails”* that specify the number of boosting iterations was set at 10.

#### Uncertainty of the classification result obtained from the single classifier

All three single classifiers employed in this research can provide the probabilistic output, (p_1_(x), ⋯, p_k_(x), ⋯, p_K_(x), k = 1, 2, ⋯, K), which can reflect the classification uncertainty of the classifier. In this paper, we used the α quadratic entropy to calculate the uncertainty [59], as in Eq (2):

$$\text{H}\left(\text{p}(\text{x})\right)=\frac{1}{\text{n}\times 2^{-2\alpha }}\sum_{\text{k}=1}^{\text{K}}{\text{p}}_{\text{k}}^{\alpha }\;\left(\text{x}\right){\left(1-{\text{p}}_{\text{k}}\left(\text{x}\right)\right)}^{\alpha }$$ (2)

where H(p(x)) represent the α quadratic entropy of the vector p(x), p_1_(x), ⋯, p_k_(x), ⋯, p_K_(x) represent the probabilistic outputs. While, α is user-defined value which ranges from 0 and 1, and α = 0.5 was chosen in this study. A smaller H(p(x)) indicates a more reliable classification. The advantage of the specificity measure is that it applies all the information in the probability vector.

#### Hybrid strategies: Voting and fusion

**Fig 4:**
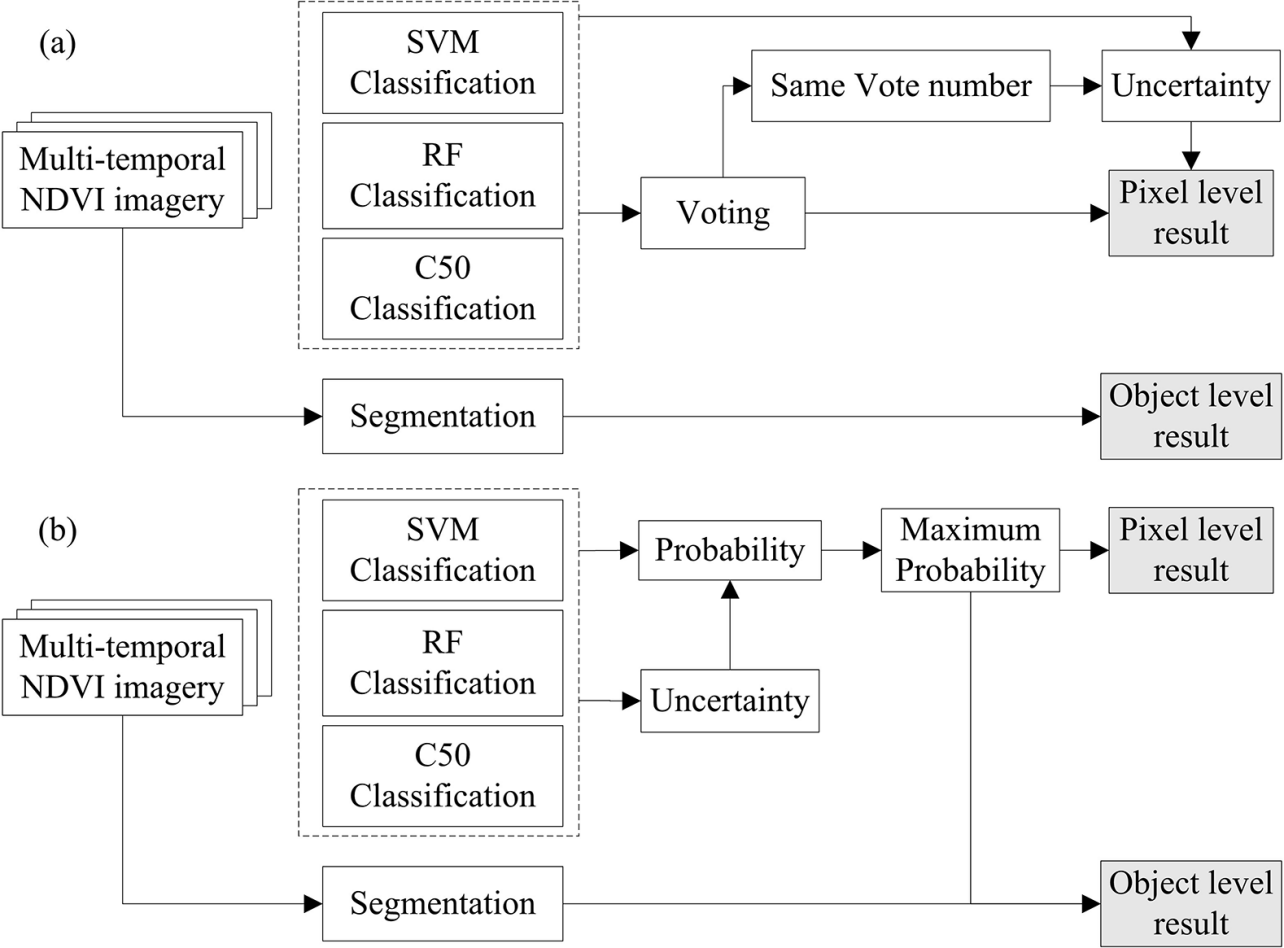
Processing chains of (a) M-voting and (b) P-fusion for the hybrid classifiers.

We used two hybrid strategies (multiple voting, M-voting and probabilistic fusion, P-fusion) to integrate single classifiers and then compared their performance with that of the single classifiers [26]. Processing chains of the voting and fusion strategies are shown in Fig 4.

In M-voting, each single classifier has a vote, and if a class obtains more votes than all the other classes, the pixel is labeled as the class with the most votes.

$$\text{C}\left(\text{x}\right)={\text{max}}_{\text{k}=\left\{1,\cdots ,\text{K}\right\}}{\text{V}}_{\text{x}}(\text{k})$$ (3)

where C(x) is the label of the pixel x and V_x_(k) is the number of votes that pixel x received for class k. Otherwise, if the votes that two or more classes obtained are the same, the classification uncertainty of the single classifiers were compared. And the pixel is labeled as the class of the single classifier with the least classification uncertainty:

$$\text{C}\left(\text{x}\right)=\text{C}\left({\text{x}}_{\hat{\text{f}}}\right)\;\text{with}\;\hat{\text{f}}={\text{min}}_{\text{f}=\left\{1,\cdots ,\text{F}\right\}}{\text{S}}_{\text{f}}(\text{x})$$ (4)

where S_f_(x) is the classification uncertainty of pixel x with classifier f, which is the H(p(x)) calculated by Eq (2) in this study. And $\hat{\text{f}}$ is the optimal classifier with the least classification uncertainty. For M-voting at the object level, a similar algorithm is employed. All pixels in an object could vote for the final label of the object, and the object is labeled as the class with the most votes as in Eq (3).

In P-fusion, the probabilistic and uncertainty results of single classifiers are utilized to define the classification label C(x), as in Eq (5):

$$\text{C}(\text{x})={\text{max}}_{\text{k}=\left\{1,\cdots ,\text{K}\right\}}\;\left\{\sum_{\text{f}=1}^{\text{F}}\frac{{\text{p}}_{\text{f}}^{\text{k}}(\text{x})}{{\text{S}}_{\text{f}}(\text{x})}\right\}$$ (5)

where ${\text{p}}_{\text{f}}^{\text{k}}\left(\text{x}\right)$ is the probabilistic value of pixel x for class k with classifier f. S_f_(x) is the uncertainty value of pixel x for classifier f, which is used to enhance the relative weight of the classification information with a low degree of uncertainty. At object level, the probabilistic and uncertainty results of all pixels in an object are used to define the label of the object. The algorithm is similar to that of pixel level (Eq (5)).

### Metric for classifier approach comparison

Along with the commonly used accuracy assessment indices, including producer’s accuracy (PA), user’s accuracy (UA), overall accuracy (OA), and kappa coefficient (kappa), calculated from the error matrix [60], McNemar’s test was employed in this study to evaluate the statistical significance of the accuracy of the different classifiers [61]. McNemar’s test is a non-parametric test based on the standardized normal test statistic, as in Eq (6):

$$\text{Z}=\frac{{\text{f}}_{12}-{\text{f}}_{21}}{\sqrt{{\text{f}}_{12}+{\text{f}}_{21}}}$$ (6)

where f_12_ is the number of samples that are correctly classified by classifier 1 and incorrectly classified by classifier 2. We defined three cases of differences in accuracy between classifier 1 and classifier 2 according to McNemar’s test:

1. No significance between classifiers 1 and 2 (N): −1.96≤Z≤1.96;
2. Positive significance (classifier 1 has higher accuracy than classifier 2) (S+): Z > 1.96;
3. Negative significance (classifier 1 has lower accuracy than classifier 2) (S-): Z < −1.96.

## Results

### Similarity Measure between Landsat and HJ NDVI

**Fig 5:**
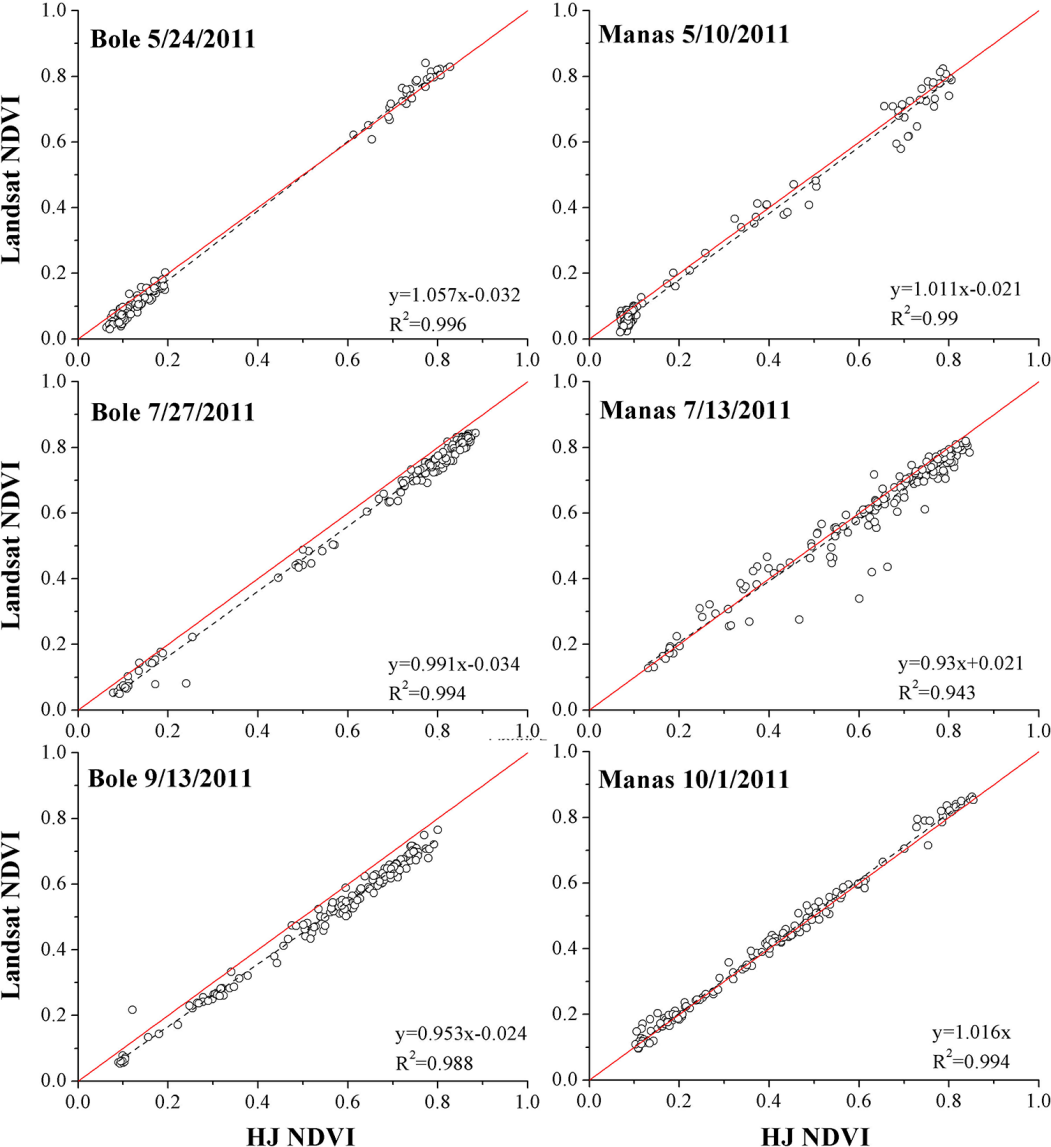
Scatter plots of NDVI data from HJ-1 CCD andLandsat-5 TM images. Note: The red lines are the 1:1 lines, and the dashed lines are fitted lines of HJ-1 NDVI and Landsat-5 NDVI.

Three matching images (acquired in May, July and September) were used to measure the consistency between Landsat-5 TM and HJ-1 CCD NDVI in both study areas. The scatter plots and linear relationships of the NDVI for the matching images are shown in Fig 5. In both study areas, the R^2^ values were larger than 0.9 for the NDVI of all the time periods. In addition, the fitted lines between Landsat-5 NDVI and HJ-1 NDVI were close to the 1:1 line. These results coincided with those of previous research and indicated that Landsat-5 TM and HJ-1 CCD data had a strong linear relationship [62–65]. Overall, the HJ-1 CCD andLandsat-5 TM images had similar spatial resolution and high NDVI consistency. Therefore, NDVI from both sensors was utilized to obtain the time series for this research.

**Fig 6:**
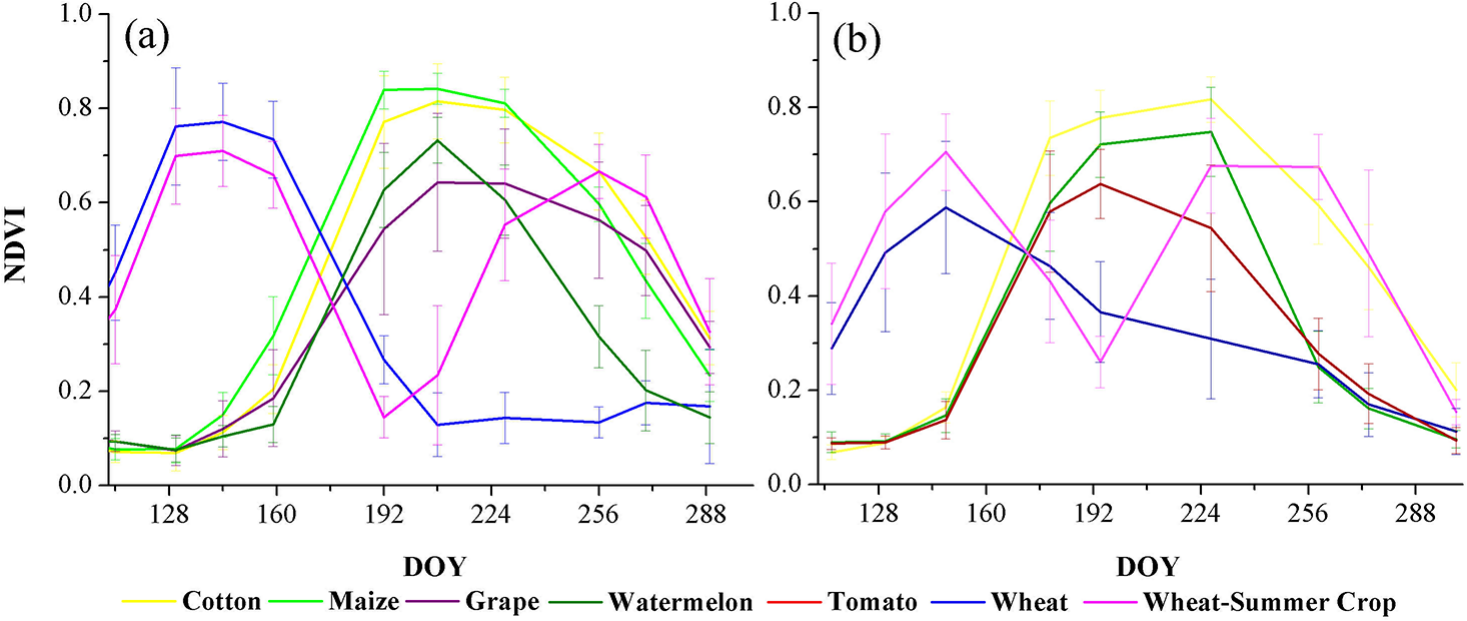
Integrated average NDVI time series of different crops in (a) Bole and (b) Manas. Note: DOY means “data of year,” and the error bars in the figures are the standard deviations of the NDVI time series of each crop type

The NDVI time series of the major crops in both study areas are shown in Fig 6. Cotton was the major crop, and the highest NDVI value of cotton was between 0.7 and 0.9 around day 200 (early August). For spring maize, the highest value was similar to that of cotton, but after the peak, NDVI of spring maize decreased faster than that of cotton, and at around day 250 (September), the NDVI of spring maize was relatively lower than the value for cotton. Similar to cotton, the NDVI peaks of tomato and watermelon were around day 200, but the NDVI values were between 0.6 and 0.7, significantly lower than those of cotton and spring maize. For grape, NDVI was high during days 170–270 (from late June to late September). In addition, the NDVI of grape had the largest variability among all crops in the study region (between 0.4 and 0.7). The major winter crop in the study areas was winter wheat, and the time period of high NDVI (above 0.5) was between day 120 and day 130.

### Accuracy assessment of classification result

The producer’s accuracy (PA), user’s accuracy (UA), and overall accuracy (OA) of the different classifiers with different training sample sizes are presented in Tables 5–8, respectively, and the results (mean and standard deviation (SD) of the accuracy) are reported based on ten runs of different training sample sets.

**Table 5:** Class-specific producer’s accuracies (PA), user’s accuracies (UA), and overall accuracies (OA) (%) for the different classifiers (Bole, training sample number = 100).

		Single Classifiers (Pixel)			Hybrid Classifiers (Pixel)		Hybrid Classifiers (Object)	
Classes		RF	SVM	C 5.0	M-Voting	P-Fusion	M-Voting	P-Fusion
Cotton	PA	93.30	89.81	88.21	91.77	91.40	94.06	92.21
		±5.63	±5.56	±4.94	±3.82	±1.88	±4.61	±4.15
	UA	70.23	69.65	69.45	71.44	71.51	70.73	70.67
		±3.58	±3.30	±2.90	±2.03	±2.93	±2.57	±1.77
Grape	PA	43.39	41.46	40.44	45.20	47.03	44.19	45.15
		±9.00	±8.72	±8.35	±7.38	±6.44	±8.79	±7.03
	UA	77.47	71.84	73.59	79.12	77.07	81.99	77.61
		±11.83	±10.81	±12.42	±8.76	±6.15	±9.97	±8.87
Maize	PA	23.51	25.39	27.34	32.24	45.32	32.82	39.29
		±19.54	±22.77	±27.13	±18.33	±15.43	±16.18	±16.30
	UA	89.84	83.77	78.07	84.91	74.51	85.03	79.41
		±14.24	±14.78	±14.32	±9.15	±8.19	±9.31	±8.67
Watermelon	PA	88.36	84.78	82.35	98.50	98.24	98.43	98.63
		±8.40	±8.43	±7.89	±0.51	±2.38	±0.61	±0.58
	UA	89.73	88.51	86.65	91.10	93.77	92.63	91.22
		±7.23	±5.85	±6.49	±3.16	±1.39	±2.95	±2.39
Wheat	PA	98.70	98.78	98.78	98.73	97.84	98.75	98.69
		±0.21	±0.17	±1.64	±0.07	±0.17	±0.18	±0.07
	UA	99.91	99.56	99.42	99.78	99.99	100.00	99.97
		±0.28	±0.67	±0.89	±0.40	±0.05	±0.00	±0.06
Wheat-summer crop	PA	99.87	100.00	100.00	99.96	95.94	99.12	99.96
		±0.12	±2.46	±12.70	±0.12	±0.00	±0.00	±0.00
	UA	97.88	91.24	88.19	96.79	98.97	99.64	99.98
		±3.92	±7.79	±8.06	±3.96	±2.07	±0.23	±0.06
OA		77.20	79.26	76.17	80.88	81.34	81.47	80.88
		±1.82	±1.80	±1.92	±0.90	±1.67	±1.11	±0.96

**Table 6:** Class-specific producer’s accuracies (PA), user’s accuracies (UA), and overall accuracies (OA) (%) for the different classifiers (Bole, training sample number = 4,000).

		Single Classifiers (Pixel)			Hybrid Classifiers (Pixel)		Hybrid Classifiers (Object)	
Classes		RF	SVM	C 5.0	M-Voting	P-Fusion	M-Voting	P-Fusion
Cotton	PA	98.44	97.60	95.01	98.15	97.28	99.52	98.44
		±0.10	±0.33	±0.58	±0.21	±0.23	±0.25	±0.19
	UA	78.39	79.30	77.19	78.90	77.21	78.89	75.71
		±0.18	±0.40	±0.40	±0.19	±0.52	±0.53	±0.17
Grape	PA	52.22	52.10	53.09	55.93	57.26	55.81	54.94
		±1.67	±4.99	±1.08	±0.52	±0.99	±0.72	±0.34
	UA	95.71	94.25	87.51	95.18	92.44	98.75	95.26
		±0.30	±0.56	±0.77	±0.55	±0.43	±0.79	±0.55
Maize	PA	86.46	80.62	85.91	88.83	85.55	92.11	91.56
		±1.16	±0.88	±2.09	±0.91	±2.45	±3.10	±2.85
	UA	98.23	96.79	90.57	97.48	95.97	100.00	100.00
		±0.34	±1.25	±2.16	±0.55	±2.15	±0.00	±0.00
Watermelon	PA	99.64	99.88	97.72	99.68	98.23	99.09	98.53
		±0.10	±0.09	±0.58	±0.13	±0.85	±0.07	±0.64
	UA	97.07	99.26	96.45	97.60	96.76	98.21	97.41
		±0.27	±0.19	±0.91	±0.46	±1.16	±0.10	±1.52
Wheat	PA	98.80	99.68	98.88	99.10	98.56	98.54	98.41
		±0.09	±0.29	0.54	±0.44	±0.16	±0.35	±0.29
	UA	100.00	99.96	99.93	99.99	99.71	100.00	100.00
		±0.00	±0.07	±0.14	±0.05	±0.37	±0.00	±0.00
Wheat-summer crop	PA	100.00	99.90	99.92	100.00	99.96	99.83	99.81
		±0.00	±0.21	±0.13	±0.00	±0.08	±0.06	±0.00
	UA	100.00	100.00	99.96	99.98	99.36	99.88	99.81
		±0.00	±0.00	±0.08	±0.06	±0.78	±0.19	±0.20
OA		87.44	87.01	86.76	89.01	89.33	89.52	88.73
		±0.56	±0.46	±0.29	±0.12	±0.26	±0.30	±0.10

**Table 7:** Class-specific producer’s accuracies (PA), user’s accuracies (UA), and overall accuracies (OA) (%) for the different classifiers (Manas, training sample number = 50).

		Single Classifiers (Pixel)			Hybrid Classifiers (Pixel)		Hybrid Classifiers (Object)	
Classes		RF	SVM	C 5.0	M-Voting	P-Fusion	M-Voting	P-Fusion
Cotton	PA	95.95	93.84	91.12	97.04	96.84	97.06	97.17
		±3.73	±4.67	±6.55	±2.38	±2.27	±2.43	±1.88
	UA	95.08	94.77	94.53	96.38	97.06	96.38	94.12
		±4.43	±4.69	±5.08	±2.53	±1.90	±2.03	±6.13
Maize	PA	79.52	78.52	78.14	83.35	80.95	85.39	86.97
		±19.35	±28.78	±11.84	±11.65	±8.93	±15.04	±8.22
	UA	79.81	77.46	76.77	83.43	84.57	85.64	80.67
		±6.57	±6.54	±8.76	±4.43	±7.44	±4.63	±6.51
Tomato	PA	65.86	63.92	60.84	75.21	78.43	79.22	76.63
		±13.62	±14.63	±13.58	±10.01	±12.41	±9.75	±12.85
	UA	81.52	76.27	75.14	79.94	76.28	81.99	78.62
		±8.68	±7.86	±10.13	±9.20	±15.82	±12.23	±6.77
Wheat	PA	80.00	79.85	85.75	84.51	73.72	81.32	74.74
		±30.62	±25.69	±28.15	±26.22	±31.73	±25.65	±30.94
	UA	94.60	83.42	77.06	96.48	99.58	98.89	98.68
		±8.89	±18.38	±24.99	±5.74	±0.70	±2.07	±2.81
Wheat-summer crop	PA	95.64	96.31	96.82	96.41	95.03	95.80	95.20
		±3.96	±3.41	±3.46	±3.62	±4.19	±3.99	±4.16
	UA	92.28	88.90	86.76	92.60	92.67	93.79	95.35
		±7.75	±7.58	±10.06	±6.49	±9.28	±6.51	±6.26
OA		91.87	92.14	91.56	93.29	92.96	93.56	93.82
		±3.15	±4.53	±4.22	±2.21	±2.61	±2.68	±2.72

**Table 8:** Class-specific producer’s accuracies (PA), user’s accuracies (UA), and overall accuracies (OA) (%) for the different classifiers (Manas, training sample number = 4,000).

		Single Classifiers (Pixel)			Hybrid Classifiers (Pixel)		Hybrid Classifiers (Object)	
Classes		RF	SVM	C 5.0	M-Voting	P-Fusion	M-Voting	P-Fusion
Cotton	PA	99.26	99.20	99.21	99.46	99.42	99.27	99.42
		±0.36	±0.16	±0.16	±0.12	±0.19	±0.12	±0.09
	UA	98.58	99.61	99.64	99.83	99.72	98.79	99.80
		±0.34	±0.13	±0.12	±0.06	±0.08	±0.06	±0.02
Maize	PA	97.04	96.75	96.90	98.01	98.60	98.11	97.24
		±1.42	±0.74	±0.86	±0.41	±0.27	±0.49	±0.16
	UA	98.89	97.36	97.46	98.31	98.02	99.84	98.23
		±1.22	±0.75	±0.64	±0.23	±0.48	±0.27	±0.24
Tomato	PA	96.20	96.15	96.29	97.71	97.54	97.17	97.54
		±1.57	±1.36	±1.00	±0.39	±0.59	±0.00	±0.18
	UA	95.46	94.58	94.94	96.29	97.54	96.01	95.19
		±1.94	±1.30	±1.08	±0.72	±0.56	±0.63	±0.16
Wheat	PA	97.44	99.81	99.85	99.96	99.62	97.37	100.00
		±0.24	±0.32	±0.26	±0.12	±0.50	±0.00	±0.00
	UA	99.31	99.03	98.77	99.85	100.00	100.00	99.85
		±0.43	±0.40	±0.35	±0.19	±0.00	±0.00	±0.19
Wheat-summer crop	PA	99.14	99.42	99.48	99.69	99.65	99.50	99.69
		±0.11	±0.13	±0.22	±0.10	±0.07	±0.12	±0.10
	UA	98.88	99.26	99.22	99.65	99.44	98.93	99.62
		±0.19	±0.23	±0.39	±0.14	±0.25	±0.00	±0.13
OA		98.27	98.36	98.42	98.99	99.04	98.68	98.80
		±0.07	±0.16	±0.18	±0.10	±0.30	±0.13	±0.16

In Bole, when the training sample number was 100, SVM obtained the highest mean OA (79.26%), C5.0 had the lowest mean OA (77.2%) among the three single classifiers; and SVM and RF had similar overall accuracy SD (around 1.8%) which was slightly lower than that of C5.0 (1.92%). Among the major crops in Bole, wheat and wheat-to-summer crop had high PA and UA (higher than 95%). However, cotton, maize, and grape had lower accuracy (UA of cotton were around 70% for each classifier, and PA of maize and grape were lower than 50% for all classifiers) because the NDVI time series these crops were confused (Fig 6). For the hybrid classifiers, both M-voting and P-fusion outperformed all single classifiers with higher mean OA (80.88% and 81.34%, respectively). The improvement occurred mainly because cotton and maize were better discriminated. At the object level, both mean OAs and OA SDs of hybrid classifiers were similar to those at pixel level. Although the accuracy of the confused crops (cotton, maize, and grape) increased when using hybrid classifiers, the accuracy remained low (for example, the mean PA of grape was 47.03% for P-fusion), which indicated that these crops were difficult to discriminate with the 100 training samples. In addition, the accuracy SDs of the hybrid classifiers were generally lower than those of the single classifiers, especially for the crops with high classification accuracy (such as wheat and watermelon). When the training sample number was 4,000, the OA of all classifiers increased, and the accuracy SD decreased significantly. Compared with the classification results obtained by using 100 training samples, the PA of both maize and grape increased significantly, but the PA of grape remained low (about 55%).

The classification accuracies of two different training sample sizes for Manas are reported in Tables 7 and 8. When the training sample number was 50, the OAs of all classifiers were around 90%. The hybrid classifiers outperformed the single classifiers (both higher OA and lower SD of OA), and the hybrid classifiers at the object level achieved similar performance as those at the pixel level. Similar to Bole, wheat-to-summer crop had high classification accuracy (both PA and UA were higher than 90% for all classifiers), and the misclassifications were due mainly to the low accuracy of maize and tomato. When 4,000 samples were used for training of the classifiers, all classifiers achieved high OA (higher than 98%) and low SD (lower than 0.2%), and the PA and UA were generally higher than 95% for all crop types.

### McNemar’s test

The McNemar’s tests for Bole and Manas are shown in Tables 9 and 10, in which the results are divided into three parts based on the training sample number. Basically, we have twelve different training sample sizes ranging from 50 to 4000 in both study areas. Then, we divided the training sample size to three groups. The training sample size ranging from 50 to 500, 750 to 2000, and 2500 to 4000 were supposed as ‘small sample size’, ‘middle sample size’ and ‘large sample size’ respectively. As we had ten model runs for each training sample size, there were 40 model runs in each group. In Bole, SVM outperformed RF, and they both outperformed C5.0; the hybrid classifiers outperformed the single classifiers as there were more “S-” in the “single classifiers versus hybrid classifiers” comparison, and M-voting outperformed P-fusion at both the pixel and object level. As for the comparison between pixel and object level, both M-voting and P-fusion had better performance at object level. For example, when the training sample number was between 750 and 2000 in Bole, “pixel-based voting versus object-based voting” had “4 S+, 23 N, 13 S-”, and “pixel-based fusion versus object-based fusion” had “17 N, 23 S-”. Additionally, when more training samples were used to train the classifiers in Bole, the number of “N” between classifier comparisons increased. For instance, there were 141 “N” when the training samples number was between 50 and 500, but there were 209 “N” when the training sample number ranged from 2,500 to 4,000. In Manas, when the training sample number was small (ranging from 50 to 500), the result of McNemar’s test was similar to that of Bole, and if more training samples were used, the classifiers had similar performances with high classification accuracy, which coincided with the result obtained in the ‘Accuracy Assessment’ section.

**Table 9:** McNemar’s test for Bole.

		Pixel based Single Classifiers		Pixel based		Object based		
		SVM	C50	M-voting	P-fusion	M-voting	P-fusion	Training sample number
Pixel based Single Classifiers	RF	2S+ 7N 31S-	40S+	40S-	40S-	40S-	2S+ 38S-	50~500
	SVM		38S+ 2N	3S+ 7N 30S-	3S+ 9N 28S-	1S+ 3N 36S-	5S+ 9N 26S-	
	C50			40S-	7N 33S-	40S-	4N 36S-	
Pixel based	M-voting				8S+ 18N 14S-	3S+ 12N 25S-	7S+ 18N 15S-	
	P-fusion					4S+ 11N 25S-	9S+ 20N 11S-	
Object based	M-voting						7S+ 14N 5S-	
Pixel based Single Classifiers	RF	6S+ 12N 22S-	40S+	40S-	1N 39S-	1N 39S-	40S-	750~2000
	SVM		40S+	3S+ 8N 29S-	5S+ 8N 27S-	3S+ 3N 34S-	2S+ 7N 31S-	
	C50			40S-	10N 30S-	40S-	3S+ 4N 33S-	
Pixel based	M-voting				14S+ 26N	4S+ 23N 13S-	1S+ 29N 10S-	
	P-fusion					7S+ 24N 9S-	17N 23S-	
Object based	M-voting						34S+ 3N 3S-	
Pixel based Single Classifiers	RF	9S+ 19N 12S-	27S+ 9N 4S-	40S-	1N 39S-	8N 32S-	40S-	2500~4000
	SVM		20S+ 18N 2S-	1S+ 1N 38S-	1S+ 4N 35S-	1N 39S-	2S+ 7N 31S-	
	C50			40S-	4S+ 9N 27S-	40S-	2S+ 18N 20S-	
Pixel based	M-voting				22S+ 18N	1S+ 23N 16S-	38S+ 1N 1S-	
	P-fusion					27N 13S-	12N 28S-	
Object based	M-voting						3S+ 33N 4S-	

Notes: The M-voting and P-fusion are implemented at both the pixel level and object level. N = No significance, S+ = Positive significance, S- = Negative significance. Classifiers in column are classifiers 1 and classifiers in line are classifiers 2 of section 3.3.

**Table 10:** McNemar’s test for Manas.

		Pixel based Single Classifiers		Pixel based		Object based		
		SVM	C50	M-voting	P-fusion	M-voting	P-fusion	Training sample number
Pixel based Single Classifiers	RF	8N 32S-	40S+	4N 36S-	1S+ 8N 31S-	1S+ 3N 36S-	7S+ 9N 24S-	50~500
	SVM		38S+ 2N	6S+ 16N 18S-	10S+ 16N 14S-	4S+ 14N 22S-	15S+ 14N 9S-	
	C50			40S-	7N 33S-	40S-	4N 36S-	
Pixel based	M-voting				15S+ 21N 4S-	3S+ 12N 25S-	22S+ 15N 3S-	
	P-fusion					6S+ 15N 19S-	16S+ 19N 5S-	
Object based	M-voting						24S+ 14N 2S-	
Pixel based Single Classifiers	RF	3S+ 25N 12S-	34S+ 6N	1N 39S-	1S+ 5N 34S-	15N 25S-	6N 34S-	750~2000
	SVM		36S+ 4N	17N 23S-	1S+ 23N 16S-	5S+ 12N 23S-	2S+ 1N 20S-	
	C50			40S-	5S+ 28N 7S-	1S+ 13N 26S-	3S+ 22N 15S-	
Pixel based	M-voting				22S+ 18N	6S+ 26N 8S-	1S+ 31N 8S-	
	P-fusion					7S+ 32N 1S-	3S+ 25N 12S-	
Object based	M-voting						2S+ 28N 10S-	
Pixel based Single Classifiers	RF	3S+ 36N 1S-	34S+ 6N	2N 38S-	7N 33S-	2S+ 21N 17S-	6N 34S-	2500~4000
	SVM		35S+ 5N	5N 35S-	11N 29S-	4S+ 7N 29S-	5N 35S-	
	C50			40S-	9S+ 28N 3S-	1S+ 28N 11S-	11S+ 27N 2S-	
Pixel based	M-voting				19S+ 21N	13S+ 17N 10S-	2S+ 37N 1S-	
	P-fusion					2S+ 21N 17S-	33N 7S-	
Object based	M-voting						8S+ 32N	

Notes: The M-voting and P-fusion are implemented at both the pixel level and object level. N = No significance, S+ = Positive significance, S- = Negative significance. Classifiers in column are classifiers 1 and classifiers in line are classifiers 2 of section 3.3.

### Classifier performance at different training sample sizes

**Fig 7:**
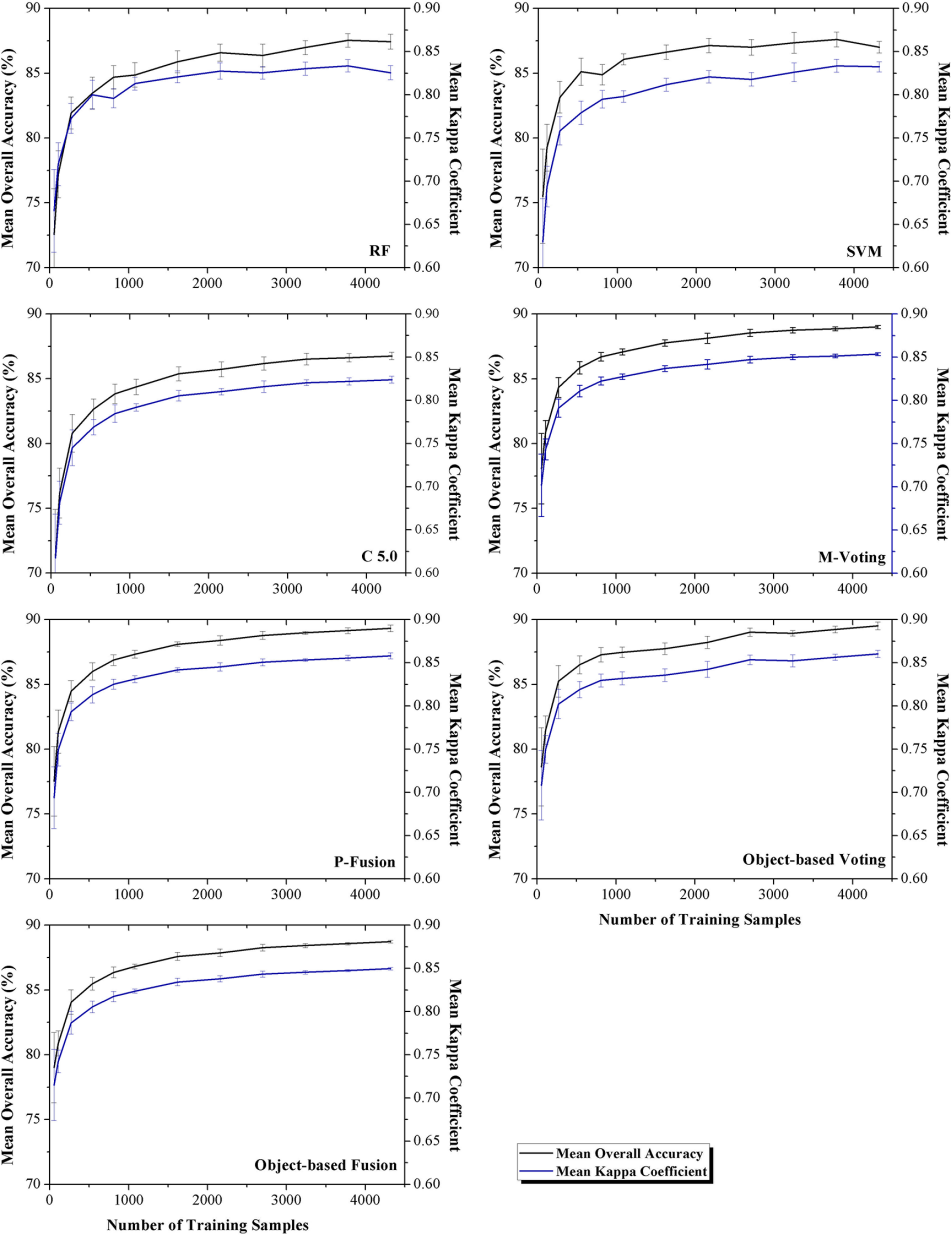
Overall accuracy of different classifiers using different training sample sets (Bole). Note: The error bars are the standard deviation of the overall accuracy.

**Fig 8:**
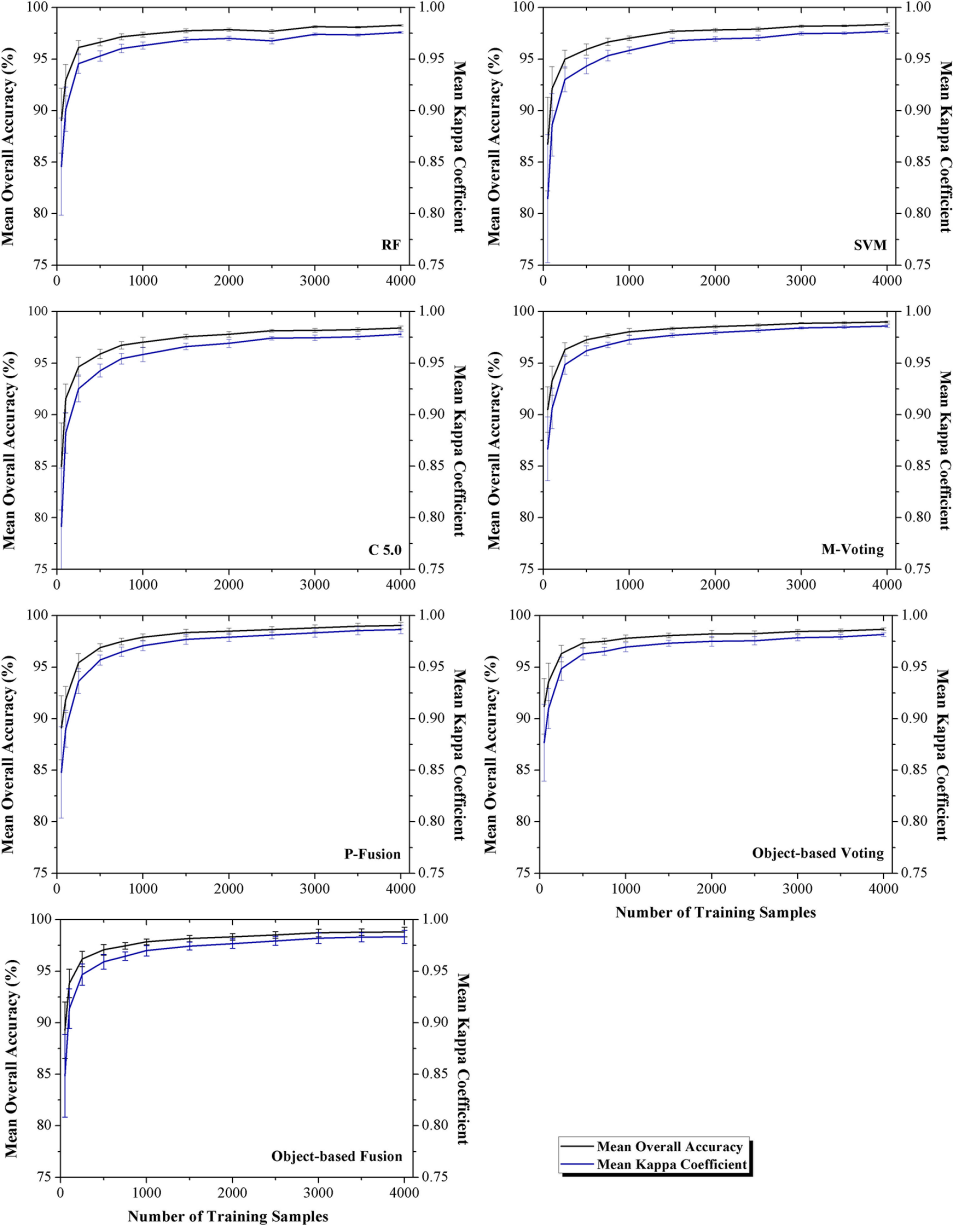
Overall accuracy of different classifiers using different training sample sets (Manas). Note: The error bars are the standard deviation of the overall accuracy.

The influences of training sample number on the classification accuracy are shown in Figs 7 and 8 for Bole and Manas, respectively. The figures indicated that the OA increased with training sample number until saturation points were reached; after that, the classification accuracy did not increase significantly. For Bole, the accuracy saturated at about 1,500 training samples. For Manas, saturation points were reached at 500 training samples. This was consistent with the accuracy assessment and McNemar’s test, which indicated that when the training sample number of Manas was larger than 500, nearly all classifiers had similar performance. In addition, the SD of the OA decreased when more samples were used to train the classifiers.

Among all classifiers, M-voting at the object level was least affected by the number of training samples, such as in Bole, the OA was above 85% when only 250 training samples were employed. Compared with the hybrid classifiers, the single classifiers were more strongly affected by the decrease of training samples. For example, when fewer than 500 training samples were utilized in Bole, the OAs of the single classifiers were lower than 85% and the accuracy SD of the hybrid classifiers was lower than that of the single classifiers. In Manas, when the training sample size was small, the situation was similar to Bole; while, when the training sample number was larger than 2000, all classifiers obtained similar low accuracy SDs.

## Discussion

In this study, the dominant crops of two representative counties, Bole and Manas, were classified using NDVI time series. For these crops, some were separable from the others crops, but some crops were confused. In Manas, for example, wheat is a winter crop, and it was well-separated from all summer crops. Thus, wheat obtained high classification accuracy (both PA and UA above 95% for nearly all classifiers), even when only 100 training samples were used (in Bole). In contrast, the NDVI time series of cotton, grape, and maize in Bole were a little confused. As shown in Fig 6, the NDVI of grape varied significantly during days 192 and 208. As a result, the NDVI profiles of some grape pixels were similar to those of cotton. In addition, some NDVI profiles series of maize were also similar to cotton. Therefore, the classification accuracy of these confused crops was relatively low. For instance, the hybrid classifiers (M-voting and P-fusion) could increase the PA of grape by 2–5%, but the PA of grape was still below 60% for all classifiers when all 4,000 training samples were utilized. Furthermore, when the crops were well-separable (such as in Manas), the saturation points of classification accuracy were reached at around 500 training samples; but if the crops were confused (such as in Bole), the classifiers needed 1,500 training samples to reach saturation points.

**Fig 9:**
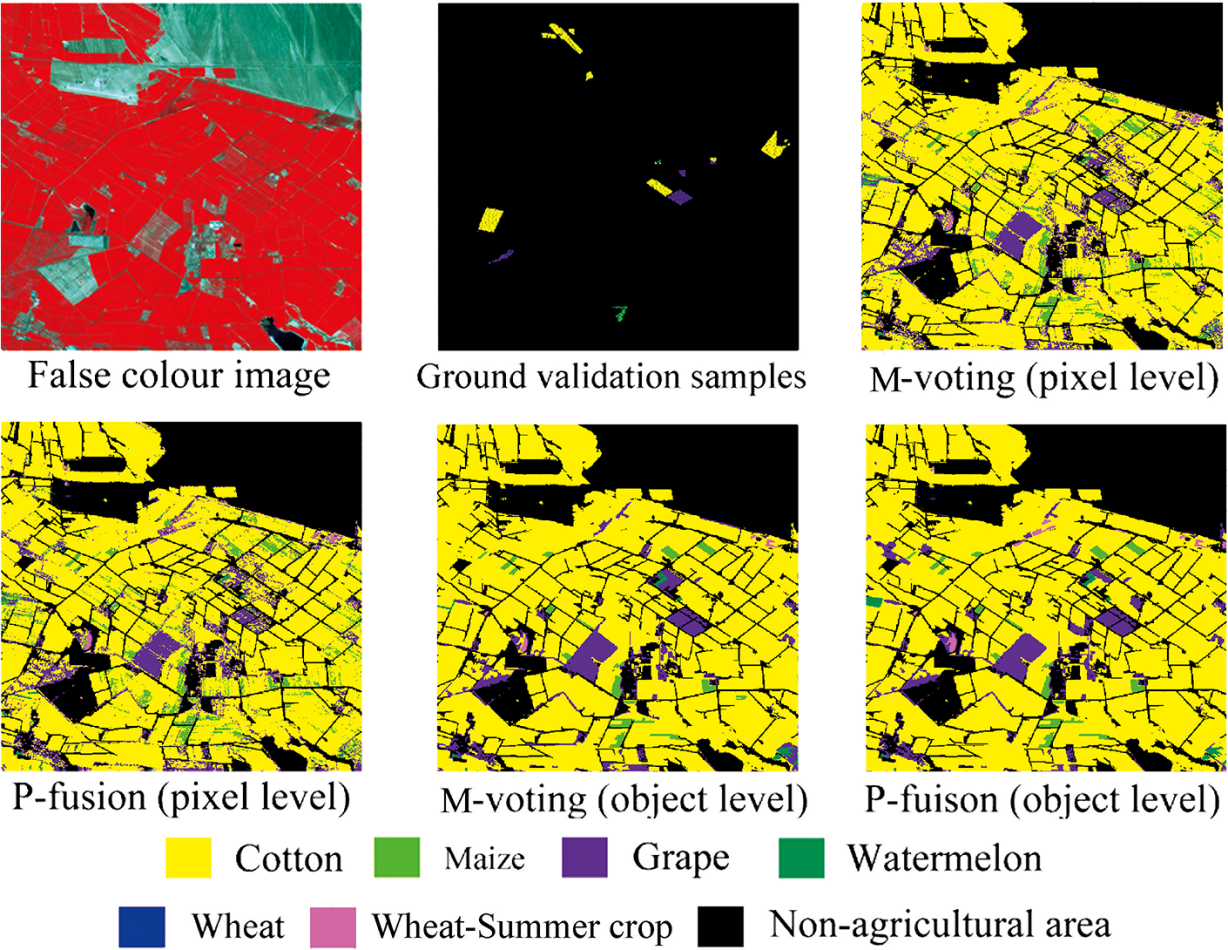
Subset image classification maps for the Bole dataset.

**Fig 10:**
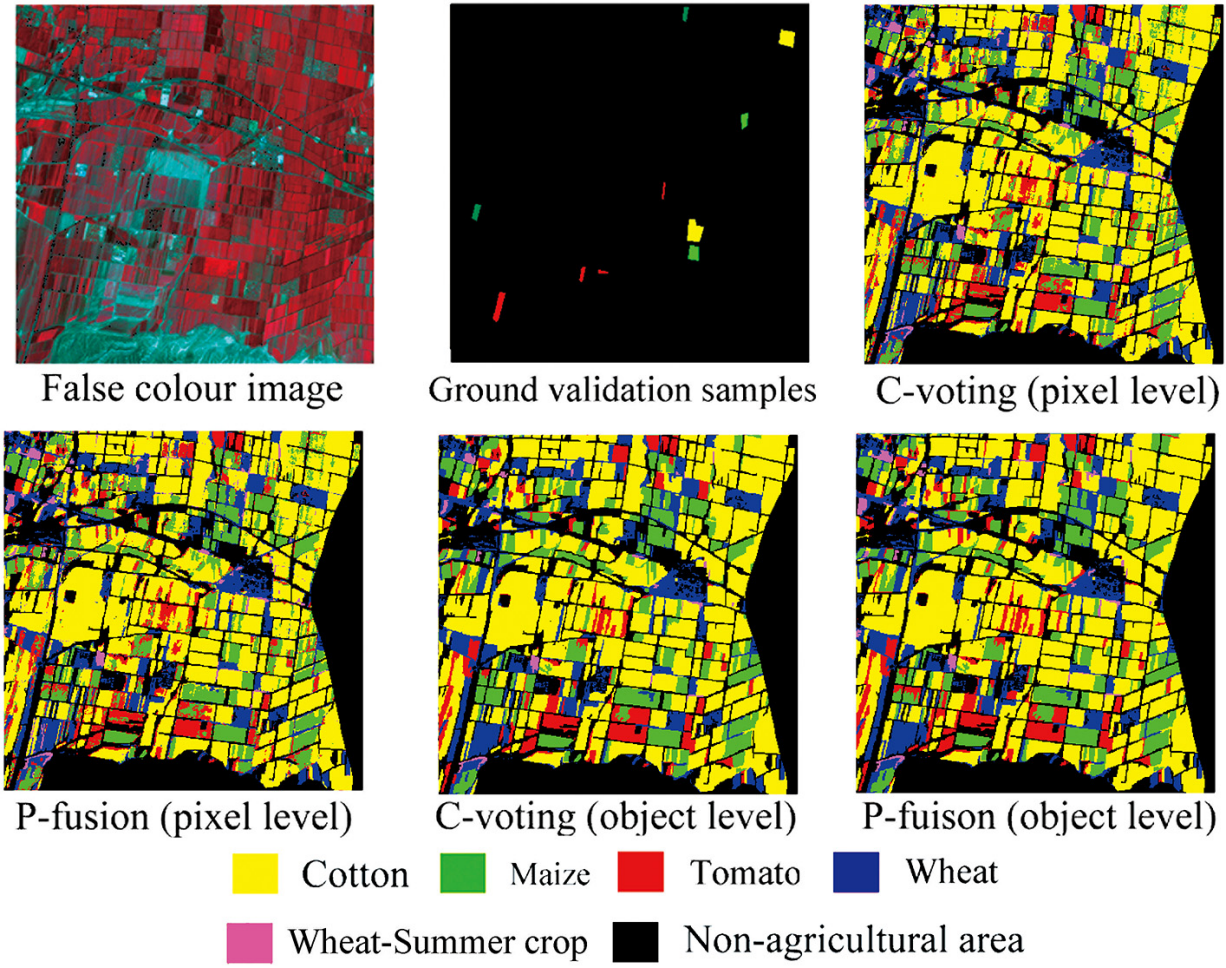
Subset image classification maps for the Manas dataset.

Between pixel-based and object-based classification, on one hand, classification accuracies were similar in both Bole and Manas. On the other hand, object-based classification provided a more visually appealing result. A series of subset images that were extracted from Bole and Manas are shown in Figs 9 and 10. Classification results for both Bole and Manas at the object level were less speckled than those at the pixel level, which was consistent with previous studies that object-based classifications could offer a more generalized visual appearance and a more contiguous depiction of land cover [34]. In addition, it is notable that both the voting and fusion methods are used at the object level, which is different from previous studies using features at object level to classify crops, and this enriches classification methods of object-based crop classification.

For different training sample size, when a small training sample set was used, the classification accuracy was low, and the hybrid classifiers could improve the classification performance substantially (Tables 9 and 10, training sample number range from 50 to 500). However, if a large training sample set was employed, single classifiers could achieve high classification accuracy (above 90%); thus, the hybrid classifiers did not improve performance significantly (Tables 9 and 10, training sample number range from 2,500 to 4,000).

Hybrid classifiers need to use the output of single classifiers, which leads to greater time consumption. However, some national and local authorities do not always pay major attention to collecting ground reference data [66], so the amount of ground reference that could be used for training the classifiers may be limited. Therefore, researchers could benefit from the hybrid classifiers when the number of training samples is small. Nevertheless, if abundant training samples are provided, single classifiers are more suitable because they can achieve high classification accuracy and are more computationally efficient than hybrid classifiers.

## Conclusion and Limitation

In this research, we employed classifier hybrid strategies, M-voting and P-fusion, to integrate single classifiers for crop classification using time series NDVI at both the pixel and object levels. The main conclusions of the research follow:

1. Landsat TM and HJ CCD have similar NDVI; thus, the two data sources could be used together to increase the temporal resolution of the NDVI time series at 30-m spatial resolution.
2. When the training sample number is small (50 or 100), hybrid classifiers outperformed single classifiers (higher classification accuracy and lower accuracy SD). Then, the larger training sample set could improve classification performance; but the improvement reaches saturation points (such as 1,500 samples for Bole and 500 samples for Manas), and additional training sample cannot further improve classification accuracy. Thus, when abundant training samples (4,000) are used, hybrid classifiers do not substantially improve classification performance compared with single classifiers.
3. OBIA did not improve the classification performance compared with the PBIA, especially in the heterogeneous region of Manas. However, OBIA can potentially solve the pixel heterogeneity problem, and fewer “salt-and–pepper” noises were observed in the classification result at the object level.

Although the hybrid classifiers can improve classification performance, especially when a small training sample set is used, the classification accuracy of some confused classes, such as grape in Bole, may remain low (e.g., less than 60%); therefore, some other features, such as texture features and physical features [14], should be used together with NDVI time series to better discriminate confused crops. Additionally, feasibility of the hybrid classifiers in some other study area should be further tested.

## Supporting Information

S1 Dataset Training and validation sample of Bole and Manas. (ZIP)
